# Digital Parenting Program: Enhancing Parenting and Reducing Child Behavior Problems

**DOI:** 10.3390/children11080980

**Published:** 2024-08-14

**Authors:** Elisa Rachel Pisani Altafim, Rebeca Cristina de Oliveira, Gabriela Aratangy Pluciennik, Eduardo Marino, Cláudia Maria Gaspardo

**Affiliations:** 1 Mental Health and Behavioral Sciences Graduate Program, Ribeirão Preto Medical School, University of São Paulo, Ribeirão Preto 14051-140, Brazil; claudiagaspardo@usp.br; 2 Independent Researcher, Ribeirão Preto 14051-140, Brazil; rebecacoliveira@gmail.com; 3 Manacá Avaliação e Aprendizagem, São Paulo 05014-000, Brazil; gabrielapluciennik@gmail.com (G.A.P.); edumarino1@gmail.com (E.M.)

**Keywords:** parenting program, positive parenting, digital parenting intervention, children’s behavior

## Abstract

Background/Objectives: Digital parenting programs using smartphone apps can support families in positive parenting and require evaluations of their effects, mainly in low- and middle-income countries with caregivers experiencing psychosocial vulnerabilities. The study evaluated the “Born Learning” digital parenting program on improving parenting practices, child prosocial behavior, and reducing the children’s externalizing behavior problems. Additionally, participants’ satisfaction and engagement with the program were evaluated. Methods: Brazilian primary caregivers of 3- to 6-year-old children totaling 91, participated in the “Born Learning” program and pre-, post-intervention, and five-month follow-up evaluations. Results: Most participants received cash transfers (64%) and reported some level of food insecurity (78%). The parenting coercive practices decreased from pre- to post-intervention, with maintenance in follow-up. Satisfaction with the role of parenting increased, and child conduct behavior problems decreased from pre-intervention to follow-up. Most participants found the program content very interesting and engaged adequately with the program strategies, such as messages and videos. Conclusions: The digital parenting program can support caregivers by enhancing parenting and decreasing child behavior problems, highlighting the potential for broader implementation in similar contexts.

## 1. Introduction

Parenting programs conducted in early childhood can positively impact parenting knowledge and practices, leading to beneficial effects on children’s development, including in low- and middle-income countries [1]. According to the “Nurturing Care” model, ensuring a child’s comprehensive development requires attention to health and nutritional needs, protection from threats such as violence, opportunities for learning, and responsive and nurturing interactions [2]. Negative parenting practices, such as spanking and verbal aggression, are high-risk factors for child development, including academic learning [3]. Therefore, supporting parents and caregivers in understanding the importance of positive and non-violent discipline is crucial for preventing violence against children, fostering positive interactions, and strengthening bonds between children and caregivers [4].

Parenting programs aimed at improving the quality of life for children and their caregivers can be conducted in formats such as group meetings, home visits, and virtually, including online programs, text messaging, regular phone calls, and mobile applications [4]. These programs that utilize technologies and the internet, such as mobile phones and messaging apps for smartphones, significantly improve parenting practices [5,6,7]. A meta-analysis showed that online parenting programs have also effectively reduced children’s behavior problems compared to control groups (e.g., waitlists) [5]. Remotely delivered parenting interventions serve as valuable complements to face-to-face programs, not replacing them but ensuring that children and their caregivers benefit from initiatives to enhance parenting and improve child outcomes [8]. As shown in a meta-analysis, technology-based parenting interventions without direct contact with an interventionist showed no evidence of effectiveness, whereas those incorporating contact were significantly more effective [6].

A universal parenting intervention delivery via WhatsApp-based groups by trained community facilitators has shown effectiveness in improving responsive caregiving and caregiver mental health in two low- and middle-income African countries, demonstrating significant potential for scalability [9]. These findings provide support for the use of digital technology programs with families experiencing psychosocial vulnerabilities [6]. Using technology in psychosocial interventions also can minimize barriers to program delivery and costs [10]. Furthermore, compared to in-person group settings, parents participating in self-directed online formats exhibited higher engagement [11].

In Brazil, parenting programs using various delivery formats, both utilizing and not utilizing technology, have been implemented and evaluated. A home visiting program, an adaptation of Reach Up for Brazil, demonstrated that mother-child dyads who completed at least ten visits improved child development [12]. The ACT Raising Safe Kids in-person group parenting program positively strengthened positive parenting practices and decreased child behavior problems [13]. When implemented in a live online group version, this same program showed an increase in parental sense of competence, and a reduction in coercive practices [14]. A video-feedback parenting program using WhatsApp called Strengthening Bonds sent personalized videos to mothers of their interactions with their children, effectively decreasing coercive practices and child behavior problems [15]. The BEM program (meaning “Brincar Ensina a Mudar” in Portuguese and translating to “Play Teaches Change” in English), an online play-based initiative delivered through WhatsApp, consisting of video classes and messages aiming to incorporate playful interactions into the daily household tasks of caregivers, showed positive impacts on improving children’s language development and decreasing intrusiveness in caregiver-child interactions [16].

The “Born Learning” (Crescer Aprendendo) Program, developed by United Way in Brazil, aims to stimulate the comprehensive development of children from 0 to 6 years by offering content and ideas for practices and activities for families conducted in partnership with philanthropic and public institutions [17]. In 2020, due to the pandemic, the program’s methodology was adapted to a virtual model using the WhatsApp platform to share content with families to support them in child development and provide emotional support while strengthening the support network among participants [18]. In 2022, building on previous experiences and considering the return of the population to in-person activities, the United Way team developed a hybrid model of the Program with two face-to-face optional meetings for families (one at the beginning and one at the end) and the delivery of daily messages, via WhatsApp for five months on the following topics: children’s rights; the role of the family; children’s physical and mental health; healthy eating; child behavior; the importance of play; violence against children and women; income generation. The main strategies of the Program were the following: (a) a WhatsApp group where daily video, audio, and text content are disseminated for exchanging information on the topic; (b) individualized sessions tailored for complex cases with potential referral to the service network; (c) both remote and in-person meetings aimed at deepening understanding of the themes; and (d) distribution of food baskets. According to the program’s records, since 2018, 10,720 families have been beneficiaries of the program. A survey conducted in 2021 by the program team found that participants reported high satisfaction with the activities offered [18].

There is a growing demand from parents regarding the practice of positive parenting. Parenting programs supported by technology can support families in positive parenting and require evaluations of their effects. Despite the “Born Learning” program being implemented in connection with public policies, there is a gap in scientific research on the program’s effects on positive parenting and child behavior. Therefore, the present study aims to evaluate the effects of the “Born Learning” parenting program on improving the parenting practices of primary caregivers of young children and child prosocial behavior and reducing the children’s externalizing behavior problems (conduct problems and hyperactivity). The study also aims to assess the participants’ satisfaction and engagement levels.

We hypothesized that coercive parenting would decrease following the intervention, while positive encouragement and parenting satisfaction would improve. Additionally, we expected that child behavior would reduce, and prosocial behaviors would increase post-intervention. It was also hypothesized that all these improvements would be maintained at follow-up.

## 2. Materials and Methods

### 2.1. Design

A single-group study with evaluations at three time points: pre-intervention, post-intervention, and follow-up, using self-report quantitative questionnaires.

### 2.2. Sample

Caregivers: 91 family caregivers from families residing in four cities within the State of São Paulo, in the Southeast of Brazil. Inclusion criterion: Primary caregivers of children aged 2 to 6 years, of both sexes, from families registered in the “Born Learning” program. Exclusion criterion: Children with any type of physical disability, mental or developmental disorder diagnosed or in the diagnostic phase.

Recruitment and contact with participants: Families were identified through the United Way registration system. The program team from the United Way informed all the program participants that researchers would contact them by phone to conduct a program evaluation. All the contacts and invitations to participate in the research were made via telephone calls or individual text messages through WhatsApp. The messages had a single sender and recipient. No lists that allowed the identification of participants or the visibility of their contact data (email, phone, etc.) to third parties were used.

A total of 160 caregivers were randomly selected according to the inclusion criteria to participate in the program evaluation, with 40 caregivers from each city from a list of 1000 participants in the “Born Learning” Program in the 2023 edition of the program. The implementation team did not have access to the names of the participants selected for the assessments during the intervention. Of these participants, 37 did not complete the post-intervention assessment due to loss of contact (n = 24), lack of interest to participate for unspecified reasons (n = 5), lack of time (n = 3), health issues (n = 2), and lack of access to a smartphone (n = 3). Then, 123 caregivers completed the intervention with pre- and post-intervention assessments. However, 32 did not complete the follow-up assessment due to loss of contact (n = 16), lack of interest to participate for unspecified reasons (n = 8), lack of time (n = 4), and health issues (n = 4). Therefore, the final sample consisted of 91 participants who completed the study with all three assessment points: pre-intervention, post-intervention, and follow-up.

Statistical analyses, including independent *t*-tests for continuous variables and chi-squared tests for categorical variables, were performed to compare the dropout participants (DP; n = 69) with those who completed the study (CP; n = 91) in order to determine if there were differences in main sociodemographic characteristics. There were no statistically significant differences regarding the child’s age and sex, or the caregiver’s skin color and schooling. There were statistically significant differences in the caregivers’ age between the DP and CP participants (DP, mean = 30.45 [±8.10]; CP, mean = 32.37 [±6.26]; *p* = 0.05). Nevertheless, the average age of both groups corresponds to young adults.

### 2.3. Ethical Aspects

Participants were informed about the study’s objectives, as well as the benefits and risks involved, and were invited to participate. After clarification and agreement to participate, participants signed the Informed Consent Form. The interviewer read the form together with the caregiver, and if the participant had any questions about the research, they were clarified. Moreover, the interviewer provided the program team’s contact phone number if the participant had any questions about the “Born Learning” Program. The research project was approved by the Research Ethics Committee of the Ribeirão Preto Medical School of the University of São Paulo (code: 69812123.4.0000.5440).

### 2.4. Instruments and Measures

The selected instruments have been translated and validated for use in Brazil and have been employed in previous studies on child development and parenting.

- Participant Sociodemographic Characterization Questionnaire: the questionnaire includes sociodemographic information about participants (e.g., age, family income, education level). The Brazilian Food Insecurity Scale [19,20] was used to characterize food insecurity situations. The scale was validated, and the cut-off point for food insecurity, according to the instrument’s guidelines, is one point, which means answering ‘yes’ to at least one question [20]. In the current research, the levels of food insecurity were not analyzed, only the dichotomous classification (presence or absence of food insecurity); therefore, three questions of the short version were used (“In the last three months, have you been concerned that the food in your house would run out before you had the means to buy, receive, or produce more food?”; “In the last three months, has the food run out before you had the money to buy more?”; “In the last three months, have you ever eaten less than you thought you should because there was not enough money to buy food?”). The parents or caregivers indicated for each statement: yes, no, or I do not know/prefer not to answer. Each yes response scores one point.
- Parenting and Family Adjustment Scale (PAFAS) [21], Brazilian version [22]. The cross-cultural adaptation of the Brazilian version included back-translation and an assessment of content validity for the instrument, followed by a confirmatory factor analysis, which demonstrated an adequate model fit, indicating that 14 items could be grouped into four subscales of the Parenting Scale [22]. The present study used the following two subscales of the Parenting Scale according to the Brazilian version, totaling seven items: Coercive Practices (4 items, ranging from 0 to 12), in which a higher score indicates more dysfunctional educational practices, and Positive Encouragement (3 items, ranging from 0 to 9), in which a higher score indicates better parenting practices. Each item is rated on a scale of the statement’s truth degree: 0 (Not at all), 1 (A little—some of the time), 2 (Quite—a good part of the time), and 3 (A lot—most of the time). The original English version showed satisfactory construct and predictive validity with a sample from Australia, demonstrating internal consistency indicators (H coefficient), of 0.78 for Coercive Parenting, and 0.75 for Positive Encouragement [21]. The PAFAS Brazilian version has been previously used in studies evaluating other parenting programs [14,15].
- Parenting Sense of Competence Scale (PSOC) [23], Brazilian Version: The scale used was the version adapted by Frontiers of Innovation (FOI) and translated by Linhares and Gaspardo [24]. This study used the satisfaction subscale, which includes nine questions on caregivers’ satisfaction with parenting [25,26]. Each item is scored on a 4-point Likert scale (from “Strongly disagree” to “Strongly agree”). The subscale total score can range from 9 to 36 points (sum of items), in which a higher score indicates more parental satisfaction. The instrument’s internal consistency for the Satisfaction subscale (English version) was 0.80 in a sample of Canadian mothers [25]. In a Brazilian study using a similar but differently translated version of the PSOC, Cronbach’s alpha was 0.75 for the satisfaction scale [27]. The PSOC FOI Brazilian version, used in the present study, has been previously used in studies evaluating other parenting programs [14,15,16].
- Strengths and Difficulties Questionnaire (SDQ) [28], translated into Portuguese and adapted to Brazilian sociocultural characteristics by Fleitlich, Córtazar, and Goodman [29]. The psychometric data on validity and reliability for the Brazilian version were described by Woerner et al. [30]. The SDQ is a screening questionnaire for child mental health problems that evaluates child behavior. The present study used subscales related to prosocial behavior and externalizing behavior problems (conduct problems and hyperactivity), totaling 15 items. The parents or caregivers indicate whether the statement is false, somewhat true, or true for each item. Responses are scored as zero, one, and two points, and for each scale, points are summed. Higher scores on the externalizing behavior problems subscale indicate more behavioral issues, while higher scores on the prosocial behavior scale signify more positive behaviors. A confirmatory factor analysis of the SDQ with a Brazilian sample demonstrated a satisfactory overall model fit and internal consistency with Cronbach’s alpha of 0.74 for externalizing behavior problems [31]. The SDQ Brazilian version has been previously used in studies evaluating other parenting programs [13,15].
- Satisfaction and Engagement Questionnaire (developed by the authors). A self-reported instrument that evaluates participant engagement and satisfaction with the program. The first five items evaluate participant engagement with the program content (e.g., reading the messages, watching the videos, completing activities) using a 4-point Likert scale (from “Never” to “Always”). Participants then rate their satisfaction with each topic covered by the program (e.g., Child behavior and emotions and ethnic-racial relations and racism) using a 5-point Likert scale (from “Not at all interesting” to “Very interesting”).

### 2.5. Procedure

- Intervention: The “Born Learning” Program is a strategy aimed at supporting the development of children in families experiencing social vulnerability, which was developed and implemented with families by the United Way team (https://unitedwaybrasil.org.br/quem-somos/no-brasil/#no-brasil; accessed on 1 August 2023). The intervention was delivered mainly remotely via WhatsApp groups over five months, with two face-to-face meetings in each municipality (one at the beginning and one at the end). The topics covered included children’s rights, the role of the family, children’s physical and mental health, healthy eating, child behavior, the importance of play, violence against children, and income generation. Each week, the intervention delivered text messages and audio and video messages on various topics. Parents received comprehensive multimedia support, including two videos per week (40 total), one weekly audio pill (20 total), and daily content messages on weekdays (100 total). The WhatsApp group remained active with daily messages from Monday to Friday and was mediated by a trained facilitator and a psychologist who interacted with the participants.

Additionally, parents were invited to participate in an optional virtual group meeting to reinforce the program’s content. From the exchange of messages, the psychologist identifies participants who need additional support and invites them to individual sessions (only eight participants of the present study participated in this support, with one to three meetings). The “Born Learning” Program also provides three food baskets to participating families during the intervention. Moreover, the team provides participants with information on connecting with public social assistance, health, and education services if necessary.

- Data Collection and Analysis: The present study was conducted in 2023. Families were invited to voluntarily participate in the “Born Learning” Program through schools and philanthropic foundations that the children attend. After the invitation, families filled out an online term of interest and registration for participation in the “Born Learning” Program. From the analysis of spreadsheets with data from the records of parents and caregivers participating in the “Born Learning” Program conducted by United Way, families with complete data were selected and invited to participate in the present research. United Way informed participants that researchers would contact them by phone for program evaluation. The interview was optional and took place voluntarily by parents and caregivers who were told that it would not interfere with their participation in the “Born Learning” Program if they did not feel comfortable answering the questionnaire.

The current research used data from three time points: pre- and post-intervention and 5-month follow-up. The families were interviewed by phone using questionnaires, including their sociodemographic characteristics, evaluations about parenting (PAFAS and PSOC) and child behavior (SDQ), and questions about participation in the program. Psychology student interns conducted telephone interviews using questionnaires under supervision.

The mean, standard deviation, and minimum and maximum values were calculated for the descriptive analysis of continuous variables. For the categorical variables, the frequency and percentage were calculated. Within-group comparisons for parenting and child behavior measures were analyzed using one-way repeated measures ANOVA to evaluate the differences between the three time points pre- post-intervention and follow-up. Pairwise comparisons were used to compare each time point to indicate any significant differences. Participant engagement and satisfaction with the program were analyzed according to their percentage. The Statistical Package for Social Sciences (SPSS, version 29.0; Chicago, IL, USA) was used for the data analysis. The level of significance was 5% for all of the tests.

## 3. Results

### 3.1. Sample Characteristics

The main sociodemographic characteristics of the caregivers and their children are shown in Table 1. The sample mainly comprised young adult mothers who had completed at least high school. The caregiver’s racial self-declaration was primarily brown “pardo” (Brazilian classification of race, according to the Brazilian Institute of Geography and Statistics, IBGE; https://www.ibge.gov.br/en/institutional/the-ibge.html, accessed on 1 August 2023). Most participants reported receiving cash transfers or other social benefits from the government. There was a prevalence of caregivers who reported experiencing some level of food insecurity. Regarding the children, there was a similar distribution between girls and boys, aged predominantly between 2 and 4 years old.

### 3.2. Parenting Practices and Parental Satisfaction

The ANOVA findings showed significant differences between moments for coercive practices and parental satisfaction (see Table 2). For coercive practices, differences were observed between pre-and post-intervention and between pre-intervention and follow-up, indicating a decrease in these behaviors immediately after the program, which was maintained at a 5-month follow-up. Regarding parental satisfaction, there were no statistically significant differences between pre- and post-intervention, but there was a significant difference between pre-intervention and follow-up, indicating an improvement in parental satisfaction over time. There were no statistically significant differences between moments in the encouragement factor (see Table 2).

### 3.3. Child Behavior

There were significant differences between moments in children’s behavioral problems. Significant differences were noticed between pre-intervention and follow-up for conduct problems, showing a decrease in these problems over time. There were no statistically significant differences between moments in the hyperactivity and prosocial subscales (see Table 2).

### 3.4. Engagement and Satisfaction

Table 3 presents the engagement of the participants with the program.

The strategies with the highest engagement were “read the messages”, followed by “watch the videos”, and “complete the suggested activities with the child”. Most participants reported that they “always” read the messages they received. Regarding the videos, at least half of the participants reported that they watched the received videos “almost always” or “always”. Interaction among program participants was minimal.

Table 4 presents data on the caregiver’s satisfaction with the program content. Most participants rated all intervention content as “interesting” or “very interesting”. The topic of “violence against children” received the highest rating, with over 80% of participants considering it “very interesting”, followed by “healthy eating” and “ethnic-racial relations and racism” (79.1% and 73.6%, respectively). Notably, only the “financial education” module was rated as “very interesting” by the minority of participants (33%). Even so, most participants found it interesting.

## 4. Discussion

This study demonstrated the positive effects of the “Born Learning” program to improve parenting practices and positively impact children’s behavior in a sample of vulnerable families. The caregivers reported good engagement and satisfaction with the program. These findings are consistent with existing literature, emphasizing the potential of technology-based parenting programs to promote positive changes in parenting behaviors [5,6,7].

The “Born Learning” program aimed to support vulnerable families by improving child development and positive parenting. Among two-thirds of caregivers received cash transfers and more than three-quarters experienced food insecurity. The provision of food baskets throughout the program may have incentivized these families to participate. This finding aligns with a previous study showing that 76% of at-risk mothers participating in a child abuse prevention program reported some level of food insecurity, highlighting the significant economic and environmental pressures these families face in their daily lives. [32] The literature has also shown that, compared to food-secure households, children in persistently food-insecure settings are almost six times more likely to experience violence [33]. Furthermore, food insecurity has shown a direct association with increased parenting stress and maternal depression [34]. These findings emphasize that families in vulnerable conditions participating in parenting programs may be experiencing various stressors, including lacking essential resources such as food, which can influence parenting. Therefore, as highlighted by the literature, combining cash transfers with parenting programs can be a valuable strategy to promote positive parenting with an impact on child development improvements [35].

In the present study, most participants reported having access to the Internet at home, and only one lacked such access. This finding is similar to a previous study that implemented a digital parental intervention to improve child development outcomes in an LMIC and found that 88% of families with young children in the community had access to smartphones with internet connectivity [36]. Technology and message programs have become popular, enabling the use of digital resources in parenting interventions in vulnerable populations [6]. Furthermore, among the barriers mentioned for participating in in-person group parental programs, participants cited the lack of support with childcare, fear of judgment from other parents in the group, and difficulties in finding time to balance work [37]. Therefore, the present study shows that technology can be an important support for providing information about child development to families.

After the program participation, the caregivers reported a reduction in coercive parenting practices that was maintained in the five-month follow-up. These findings support the results in the literature indicating the potential of parental interventions to reduce negative parenting practices, such as physical punishment, harsh discipline, and coercive practices [38], even in programs that use remote delivery strategies [5,7]. This result aligns with a study of Chinese mothers who participated in an 8-session virtual group intervention, which reported reduced use of corporal punishment, emotional abuse, and overall maltreatment compared to the control group [39]. They are also in line with the findings of the ACT program implemented remotely in the United States, which found a decrease in verbal and physical punishment after participation in live group meetings [40]. Interestingly, the content that was rated the highest by the participants was child abuse. Through parental programs, it is possible to sensitize parents about changes in beliefs about parenting and their perception of their children, including understanding their role in child behaviors [37].

Differently, satisfaction with the parental role showed no significant immediate changes between pre-and post-intervention but improved significantly at the follow-up compared to the previous assessments. This finding might reflect the caregivers’ gradual internalization and use of the program’s content. Furthermore, it is possible that when the caregivers recognized the changes in their children’s behavior, which were also observed in the follow-up, they felt more confident. As highlighted by the literature, parents recognized the positive impacts of the interventions in improving their relationship with their children, and they felt more confident in dealing with their children and more satisfied with their roles as caregivers [37]. This finding supports the program strategies that learning about fundamental aspects of child development could help caregivers manage challenging behaviors, which contributed to changing their perceptions of their children and improving parental satisfaction. Similar to the present study, research on another parenting program, the Family Education and Support, implemented through a community-based approach in an in-person format, also demonstrated increased parental satisfaction among participants in an African country [41].

The results showing improvements in children’s conduct behavior problems at follow-up are aligned with previous studies that demonstrated the effectiveness of digital parental interventions in reducing challenging behavior problems [5]. Considering the time needed for change, the finding is aligned with a study using the low-intensity online version of the Triple P program, which also showed delayed improvement in children’s behavioral issues, observed only at the 9-month follow-up [42]. However, the present findings differ from face-to-face programs such as the ACT Raising Safe Kids program [13] and remote video coaching interventions like Strengthening Bonds [15], which reported immediate improvements in child behavior following the intervention. This need for a longer duration in perceived changes suggests that the WhatsApp-based content delivery model of the “Born Learning” program may require more time for the effects to become evident. One hypothesis is that these other programs using different delivery methods may provide more immediate feedback and reinforcement, resulting in quicker behavioral changes in children. However, the content offered in the “Born Learning” program, while not personalized, focuses on increasing parental knowledge, leading to improved parenting practices. Consequently, this approach may result in more medium-term changes in children’s behavior. Gradually disseminating information and strategies through WhatsApp might encourage sustained learning and the gradual application of positive parenting practices. Therefore, the duration and continuity of support should be considered in the design of parenting interventions. Future research should explore how different delivery methods influence the timing and sustainability of behavioral outcomes in parenting programs.

One hypothesis for the lack of changes regarding the child’s hyperactivity behavior is that the program’s content and strategies were not specifically tailored to address hyperactivity but focused more on general parenting skills and child development. Additionally, the “Born Learning” program is a universal prevention program, and as demonstrated in the literature, indicated that treatment interventions have stronger effects on reducing disruptive child behaviors [43]. Furthermore, a Triple P online program study demonstrated stronger effects on reducing behavioral problems when participants received clinical support compared to those completing the self-directed program [44]. The “Born Learning” program offers support through optional sessions with a psychologist, although in the current sample, a very low number of participants used this strategy. Therefore, strengthening this strategy may be essential for achieving more effective outcomes in children’s behaviors, needing further investigation.

The findings also showed no significant changes regarding the child’s prosocial behavior and positive encouragement. However, the caregivers already report at baseline high scores for both measures; therefore, this lack of changes can be due to a “ceiling effect”. The present findings are similar to a previous study that used a video feedback program delivered remotely among mothers of children with and without behavior problems, which found a reduction in behavioral problems after the intervention but did not show improvement in the prosocial behavior of children who already had high values close to the maximum [45]. Regarding positive encouragement, a ceiling effect also may be present, but this needs further investigation. A study on the group-based ACT program, implemented online, showed similar findings to the current research, with a decrease in coercive practices but no change in positive encouragement, which was high in the pre-intervention and slightly above the average score of the present study [14].

The participants highly rated the program’s content. Among the topics, ‘violence against children’, ‘ethnic-racial relations and racism’, ‘healthy eating’, ‘general health’, and ‘child development’ were notable, with more than 70% of participants rating them as ‘very interesting’. This finding aligns with the nurturing care framework and global priorities in early childhood development, emphasizing the need for comprehensive approaches that address various aspects of child well-being [2]. Furthermore, the program’s emphasis on diverse topics relevant to child development and family well-being highlights the importance of addressing various issues through parenting interventions. The program mainly uses standardized content provided by a structured curriculum, which increases the potential for replicability and scalability of the intervention.

Ethnic-racial relations and racism were the third most interesting topics for participants, demonstrating the necessity of addressing this issue alongside child violence, which was considered the most crucial topic. Including this topic can be considered an innovation in the field of parenting programs, aligning with contemporary efforts to promote social equity and cultural competence within family dynamics. The literature highlights that parent-involved ethnic and racial socialization programs are powerful strategies for supporting children who experienced racial and ethnic discrimination, given parents’ crucial roles in socializing their children [44]. Furthermore, a literature review showed that despite the expanding scientific literature on the relations between ethnic and racial socialization and child development, few studies have investigated the effects of programs addressing this topic [46]. It is noteworthy that in the present study, most participants were black or “pardo” (as called in Brazil), which may be related to their interest in the topic due to the ethnic-racial relations issues experienced by this population.

The majority of participants consistently engaged with the content, indicating that the program was well-received and effectively implemented. This finding corroborates the previous satisfaction survey of the program, which also showed a high level of satisfaction with the activities offered [18]. The flexibility of the program, allowing participants to receive messages and videos and view them at their convenience, may be linked to their satisfaction with the program and the content presented. The literature shows that interventions that offer flexibility in accessing the content may lead to higher satisfaction and acceptability in digital parental programs, promoting ongoing engagement with the intervention [47]. However, in the present study, a significant portion (36%) reported not doing or rarely engaging in the suggested activities with their children. Therefore, the program should consider better ways to engage caregivers in these activities.

The current study also presents some limitations that should be acknowledged. Firstly, the pre-post-follow-up design lacks a control group, limiting the ability to make causal inferences regarding the program’s effectiveness. Therefore, it is challenging to determine if changes in parenting and child outcomes can be attributed solely to the intervention or if other external factors may have influenced the results. However, a systematic review of parenting programs and violence prevention [38] found that half of the studies utilized single-group designs, and only a small proportion (33%) employed randomized controlled trials (RCTs). Therefore, according to the literature, while using RCTs is ideal for establishing program efficacy, single-group designs are commonly used in this field due to practical and cost issues, especially in low- and middle-income countries [48]. Secondly, since the data collection was conducted via the phone interview limited to 20 to 30 min to avoid participant fatigue, only the most relevant instrument subscales related to the program were included, potentially missing other essential aspects of the participants’ experiences and outcomes, such as parental inconsistency and child internalizing behavior problems. Also, there were no direct measurements of the child and mother’s behaviors. Therefore, another limitation is the use of only self-reported data, which may be subject to social desirability biases. Participants’ responses regarding their experiences and outcomes may be influenced by their desire to present themselves in favorable parenting. However, as demonstrated by the literature, most parenting program studies rely on self-report measures [5,38]. Another limitation was the 43% loss in respondents from pre-assessment to follow-up, which may bias the retention towards participants who reported changes in their practices and were satisfied with the program. However, it was found that the main reason for this loss was difficulty in maintaining contact with these participants, as they may lose connection after the program, which could also be related to difficulties in ongoing participation and potential challenges in adherence to the data collection methods employed. Additionally, it was found that the participants who dropped out of the study and those who completed it had similar sociodemographic characteristics, with the only difference being the caregivers’ age. Despite the dropout participants being slightly younger on average, the mean age of both groups falls within the young adult stage. Therefore, the findings suggest that the groups were comparable.

Despite these limitations, the present study provides valuable findings into the potential benefits of the “Born Learning” program and highlights the need for continued evaluation of parenting programs. Future research should use RCT to strengthen the evidence base for the program’s effectiveness. Additionally, larger sample sizes and more diverse populations should be considered to enhance the generalizability of the findings. Future studies may also explore the program’s effects on other outcomes it addresses, such as play, healthy eating, and ethnic-racial relations.

## 5. Conclusions

In conclusion, the results of the present study provide preliminary scientific evidence of the potential of the “Born Learning” digital parenting program to improve positive parenting by decreasing coercive practices after the intervention and, in the medium term, strengthening satisfaction with parental role, which was related to a decrease in child conduct behavior problems. These improvements are essential for preventing violence against children, strengthening the relationships between children and caregivers, and promoting child development.

Given that most participants faced food insecurity, linking the parenting program with cash transfer programs and other support initiatives for vulnerable populations could be a strategy to strengthen caregivers’ and children’s well-being. The majority of participants consistently engaged with content focused on violence against children, ethnic-racial relations and racism, healthy eating, general health, and child development. This consistent engagement suggests that the program was well-received and the importance of parenting programs addressing these topics.

## Figures and Tables

**Table 1:** Sociodemographic characteristics of the sample.

Characteristics of the Sample	Total (n = 91)
**Child**	
Sex—girls—f (%)	55 (60.4)
Age (years)—mean (SD; range)	4.0 (1.41; 2–6)
Age groups (years)—f (%)	
2–4 years	54 (59.3)
5–6 years	37 (40.7)
**Caregiver–f (%)**	
Mother	89 (97.8)
Others (father and stepmother)	2 (2.2)
Caregiver’s Age (years)—mean (SD; range)	32.4 (6.26; 20–46)
Caregiver’s skin color ^a^—f (%)	
White	25 (27.5)
Brown	46 (50.5)
Black	20 (22)
Caregiver’s schooling—f (%)	
With no formal education	1 (1.1)
Incomplete elementary education	21 (23.1)
Elementary school	12 (13.2)
High school	49 (53.8)
Undergraduate degree	8 (8.8)
City—f (%)	
Campinas	27 (29.7)
Louveira	20 (22.0)
São Bernardo do Campo	25 (27.5)
São Paulo	19 (20.8)
Government social benefits	
Cash transfer—f (%)	58 (63.7)
Others (food, gas)	7 (7.7)
No	26 (28.6)
Food insecurity	
Yes	71 (78)
Access to the internet at home	
Yes	90 (98.9)

Note. SD = standard deviation; f = frequency; % = percentage; n = number of participants; ^a^ The classification of race/ethnicity in the study followed the official classification of the Brazilian Institute of Geography and Statistics (IBGE) that used the skin color: white, black, brown, indigenous or yellow https://www.ibge.gov.br/en/institutional/the-ibge.html, accessed on 1 August 2023.

**Table 2:** Parenting practices, satisfaction with parenting role, and child behavior (pre-, post-intervention, and follow-up).

Parenting Practices, Sense of Competence, and Child Behavior	Pre-Intervention Mean (SD)	Post-Intervention Mean (SD)	Follow-Up Mean (SD)	*p*-Value	Contrast between Times
**Parenting Practices (PAFAS)**					
Coercive practices	3.70 (1.62)	3.20 (1.54)	3.23 (1.59)	0.01	Pre > Post; Pre > Follow up
Positive Encouragement	7.33 (1.43)	7.29 (1.45)	7.51 (1.33)	0.31	NS
**Parenting Sense of Competence (PSOC)**					
Satisfaction	24.27 (3.67)	24.44 (3.26)	26.97 (5.37)	<001	Pre < Follow-up; Post < Follow-up
**Child’s behavior (SDQ)**					
Conduct problems	3.52	3.03	2.87	0.01	Pre > Follow-up
Hyperactivity	4.04	3.96	3.80	0.58	NS
Prosocial behavior	8.65 (1.47)	8.79 (1.59)	8.99 (1.30)	0.08	NS

Note: SD = standard deviation; NS = no significance; PAFAS = Parenting and Family Adjustment Scale; range score: coercive practices = 0–12; Higher scores indicated more coercive practices; positive encouragement = 0–9; higher scores indicated more encouragement; PSOC = Parenting Sense of Competence Scale—range score: satisfaction = 9–36; SDQ = Strengths and Difficulties Questionnaire—range score for each scale 0–10 each. Higher scores indicated more behavioral problems (conduct problems and hyperactivity), and prosocial behavior scale; higher scores indicated greater capacity.

**Table 3:** Participant engagement with the program (n = 91).

Participant Engagement with the Program	Never	Rarely	Sometimes	Almost Always	Always
f (%)					
Read the messages	0	10 (11.0)	4 (4.4)	14 (15.4)	63 (69.2)
Watch the videos	2 (2.2)	23 (25.3)	11 (12.1)	25 (27.5)	30 (33)
Complete the suggested activities with the child (e.g., play and tell a story)	8 (8.8)	25 (27.5)	14 (15.4)	19 (20.9)	25 (27.5)
Reply to messages	17 (18.7)	21 (23.1)	18 (19.8)	14 (14.3)	22 (24.2)
Interact with other participants and comment on messages	38 (41.8)	17 (18.7)	19 (20.9)	7 (7.7)	10 (11)

Note. f = frequency; % = percentage; n = number of participants.

**Table 4:** Participant satisfaction with program content (n = 91).

Participant Satisfaction with Program Content	Not at All Interesting	Slightly Interesting	Somewhat Interesting	Interesting	Very Interesting
f (%)					
Violence against children	1 (1.1)	1 (1.1)	1 (1.1)	13 (14.3)	75 (82.4)
Healthy eating	1 (1.1)	0	1 (1.1)	17 (18.7)	72 (79.1)
Ethnic-racial relations and racism	2 (2.2)	0	1 (1.1)	21 (23.1)	67 (73.6)
General Health	1 (1.1)	0	3 (3.3)	22 (24.2)	65 (71.4)
Child development	0	2 (2.2)	3 (3.3)	21 (23.1)	65 (71.4)
Accidents at home	3 (3.3)	2 (2.2)	7 (7.7)	17 (18.7)	62 (68.1)
Child behavior and emotions	1 (1.1)	0	1 (1.1)	29 (31.9)	60 (65.9)
Plays	0	1 (1.1)	4 (4.4)	26 (28.6)	60 (65.9)
Music and storytelling	1 (1.1)	0	6 (6.6)	29 (31.9)	55 (60.4)
Financial education	1 (1.1)	6 (6.6)	5 (5.5)	49 (53.8)	30 (33.0)

Note. f = frequency; % = percentage; n = number of participants.

## Data Availability

The data can be made available for consultation from the corresponding author upon request, but restrictions apply to their availability according to the ethical approval. The data are not publicly available due to privacy and ethical restrictions.
